# Prior Cardiovascular Treatments—A Key Characteristic in Determining Medication Adherence After an Acute Myocardial Infarction

**DOI:** 10.3389/fphar.2022.834898

**Published:** 2022-03-07

**Authors:** Anna Campain, Carinna Hockham, Louisa Sukkar, Kris Rogers, Clara K Chow, Thomas Lung, Min Jun, Carol Pollock, Alan Cass, David Sullivan, Elizabeth Comino, David Peiris, Meg Jardine

**Affiliations:** ^1^ The George Institute for Global Heath, UNSW, Sydney, NSW, Australia; ^2^ Faculty of Medicine, University of New South Wales, Sydney, NSW, Australia; ^3^ School of Public Health, Imperial College London, The George Institute for Global Health, London, United Kingdom; ^4^ Faculty of Medicine and Health, The University of Sydney, Sydney, NSW, Australia; ^5^ Graduate School of Health, University of Technology Sydney, Sydney, NSW, Australia; ^6^ Westmead Applied Research Centre, Faculty of Medicine and Health, University of Sydney, Sydney, NSW, Australia; ^7^ Department of Cardiology, Westmead Hospital, Sydney, NSW, Australia; ^8^ Renal Division, Kolling Institute for Medical Research, Sydney, NSW, Australia; ^9^ University of Sydney, Sydney, NSW, Australia; ^10^ Menzies School of Health Research, Charles Darwin University, Darwin, NT, Australia; ^11^ Department of Chemical Pathology Royal Prince Alfred Hospital, Camperdown, NSW, Australia; ^12^ NSW Health Pathology, Newcastle, NSW, Australia; ^13^ Central Clinical School, University of Sydney, Camperdown, NSW, Australia; ^14^ NHMRC Clinical Trials Centre, The University of Sydney, Sydney, NSW, Australia; ^15^ Concord Repatriation General Hospital, Sydney, NSW, Australia

**Keywords:** medication adherence, acute myocardial infarction, AMI, linked data, big data, routinely collected data, cardioprotective medications

## Abstract

**Objective:** To investigate long-term adherence to guideline-recommended cardioprotective medications following hospitalization for an acute myocardial infarction (AMI), and identify characteristics associated with adherence.

**Methods:** An Australian population-based cohort study was used to identify participants who had their first AMI between 2006 and 2014 and were alive after 12 months. Linked routinely collected hospital, and prescription medication claims data was used to study adherence over time. Predictors and rates of adherence to both lipid-lowering medication and renin-angiotensin system blockade at 12 months post-AMI was assessed.

**Results:** 14,200 people (mean age 69.9 years, 38.7% female) were included in our analysis. At 12 months post-AMI, 29.5% (95% CI: 28.8–30.3%) of people were adherent to both classes of medication. Individuals receiving treatment with both lipid-lowering medication and renin-angiotensin system blockade during the 6 months prior to their AMI were over 9 times more likely to be adherent to both medications at 12 months post-AMI (66.2% 95% CI: 64.8–67.5%) compared to those with no prior medication use (treatment naïve) (7.1%, 95% CI: 6.4–7.9%). Prior cardiovascular treatment was the strongest predictor of long-term adherence even after adjusting for age, sex, education and income.

**Conclusions:** Despite efforts to improve long-term medication adherence in patients who have experienced an acute coronary event, considerable gaps remain. Of particular concern are people who are commencing guideline-recommended cardioprotective medication at the time of their AMI. The relationship between prior cardiovascular treatments and post AMI adherence offers insight into the support needs for the patient. Health care intervention strategies, strengthened by enabling policies, are needed to provide support to patients through the initial months following their AMI.

## Introduction

Cardiovascular disease (CVD) remains the leading cause of death globally, despite considerable advances in effective preventive treatments. It is estimated that cases of CVD have nearly doubled between 1990 and 2019, with estimates reaching 523 million prevalent cases in 2019 (Roth et al., 2020). Acute myocardial infarction (AMI) accounts for almost half of CVD-related deaths globally (Roth et al., 2017). Based on clear evidence of benefit from large-scale randomized controlled trials, all international guidelines recommend long-term secondary prevention medications for patients who have had an AMI, unless contraindicated (Guidelines for the Management of Acute Coronary Syndromes, 2006; Smith et al., 2006; National Heart Foundation of Australia and the Cardiac Society of Australia and New Zealand, 2012; National Vascular Disease Prevention Alliance. Guidelines for the management of absolute cardiovascular disease risk, 2012; Roffi et al., 2016; Ibanez et al., 2017; Karmali and Lloyd-Jones, 2017). These include both lipid-lowering and blood pressure-lowering medications. Relative risk reductions in subsequent coronary events are estimated to be around 20% for every 10 mmHg reduction in blood pressure (Karmali and Lloyd-Jones, 2017) and 24% for every 1 mmol/L decline in low density lipoprotein (LDL) cholesterol (Armitage et al., 2019; Yusuf et al., 2000). Despite this compelling evidence, gaps in recommended medication use of up to 50% have been observed. (Sabate, 2003; Heeley et al., 2010; Hall et al., 2016).

Multiple factors associated with sub-optimal medication adherence in CVD have been identified. These range from patient characteristics (e.g., age, sex (Hall et al., 2016) and education (Shang et al., 2019)) to health system factors (e.g., medication cost and healthcare access (Sabate, 2003)) and provider factors (e.g., failure to prescribe, up-titrate or re-commence guideline-based medications (Heeley et al., 2010)). Psychosocial and psychological associations (e.g., patient’s belief and attitudes) with adherence have also been addressed separately (Molfenter et al., 2012). There is little consistency among results (Gast and Mathes, 2019; Leslie et al., 2019) leading to difficulties identifying targets for interventions.

As research evolves from small and carefully curated data sets to large and expansive data, our understanding of the influencers of medication adherence has the opportunity to grow (Kardas et al., 2020). Large, complex and longitudinal data sources are emerging and over the last decades, more hospital administration data are becoming available for research purposes along with pharmaceutical dispensing data. Both these data sources are often developed for cost and budgeting purposes but can be used for health service research to inform clinical and pharmacoepidemiologic research (Nicholls et al., 2017). Longitudinal survey data including the Nurses’ Health Studies (Belanger et al., 1978), the 45 and Up Study (45 and Up Study Collaborators, 2008), the 1970 British Cohort Study (Elliott and Shepherd, 2006) and the Millennium Cohort study (Connelly and Platt, 2014) all follow large populations over time gaining insights into participants’ health and social characteristics.

Alongside increases in the availability of these rich data sources, there has been an expansion in the past 15 years of machine learning and advanced statistical methods with which to analyse such data. These advanced methods are being applied more often in a wider scientific context and in recent years these methods have been instrumental in the medication adherence paradigm (Zullig et al., 2019; Gu et al., 2021).

A more comprehensive understanding of the factors associated with adherence is needed to address treatment gaps. In this study, we use advanced statistical methods and big data to investigate adherence in people hospitalised with a first AMI. Using data from a large cohort study involving survey data, routinely collected hospital administrative and pharmaceutical dispensing data in Australia, we aimed to: 1) examine adherence over time to both a lipid-lowering medication and renin-angiotensin system (RAS) blockade post-AMI; 2) identify factors associated with adherence to both medication classes in combination; and 3) assess the strength of these associations using advanced regression methods.

## Methods

### Study Context

This study uses data from the 45 and Up Study and the EXamining ouTcomEs in chroNic Disease in the 45 and Up Study (EXTEND45) Study. Details of both the 45 and Up Study (45 and Up Study Collaborators, 2008; Banks et al., 2011) and EXTEND45 (Foote et al., 2020) have been published previously. In summary, the 45 and Up Study is an Australian population-based cohort study of 267,153 men and women aged ≥45 years who were randomly sampled from the general population of New South Wales (NSW), using the Services Australia (formerly Department of Human Services) enrolment database.

Between 2006 and 2009, invited participants were asked to complete a postal questionnaire on healthy ageing and consent to ongoing linkage to their data held in routinely collected databases. The 45 and Up Study had an 18% response rate covering approximately 11% of the NSW population aged 45 years and over (45 and Up Study Collaborators, 2008) and has been shown to report near representative estimates for many of the various measures relating to risk factors estimated by the NSW health survey (Mealing et al., 2010). In the EXTEND45 Study, 45 and Up Study participants and their baseline questionnaire responses have been linked to routinely collected administrative health datasets, outpatient laboratory results from laboratory service providers, and the Australia and New Zealand Dialysis and Transplant (ANZDATA) registry.

### Ethics Approval

The EXTEND45 Study received ethical approval from the NSW Population and Health Services Research Ethics Committee (PHSREC; study reference number HREC/13/CIPHS/69). The 45 and Up Study received ethical approval from the University of New South Wales Human Research Ethics Committee (HREC).

### Data Sources

The linked data sources used within this work include 1) NSW Admitted Patient Data Collection (APDC), providing information on all public and private hospital admissions in NSW, 2) Medicare Benefits Schedule (Department of Health, Australian Government, 2021a) (MBS) database, providing information on government-subsidized medical services, 3) Pharmaceutical Benefits Scheme (Department of Health, Australian Government, 2021b) (PBS) database, an electronic dispensing record providing prescription medication claims data, 4) community laboratory services, and 5) NSW Register of Births, Deaths and Marriages (RBDM). MBS and PBS data were provided by Services Australia through a deterministic link with 45 and Up Study participants. Probabilistic linkage of all other data sources was performed by the Centre for Health Record Linkage (CHeReL) (http://www.cherel.org.au).

### Study Cohort

Participants were included in the present study if they were hospitalized with their first AMI between 1st January 2006 and 1st October 2013 (hereafter referred to as the index AMI). Hospitalization records and self-reported results were used to validate an incident AMI. Further details of the selection criteria and study cohort are available in the supplementary material.

Follow-up lasted from the date of AMI hospitalization discharge until 30th June 2014 (end of available data). Participants were censored at a second AMI or death and required at least 9 months of follow up. AMI diagnoses were identified using the International Statistical Classification of Disease and Related Health Problems, 10th Revision, Australian Modification (ICD-10-AM) codes (Supplementary Appendix Supplementary Table S1).

### Covariates

Covariates included demographic, socioeconomic, lifestyle and clinical characteristics, and were derived from either self-reported information from the 45 and Up Study baseline questionnaire, PBS, MBS or laboratory data, or a combination of these. Supplementary information for covariates and Appendix Supplementary Table S2 contains further details.

The primary exposure of interest was prior treatment with a lipid-lowering medication and/or RAS blockade (defined below), which was identified from PBS data, using a 6-months lookback interval from the index AMI (hereafter referred to as prior treatment exposure). The term “exposure” rather than “adherence” is used in the pre-AMI time period because guideline-based indications for the individual prior to the AMI cannot be ascertained in the dataset. Four mutually exclusive classes of prior treatment exposure were defined:

(1) treatment naïve, neither a lipid-lowering medication nor RAS blockade;
(2) a lipid-lowering medication but no RAS blockade;
(3) RAS blockade but no lipid-lowering medication; and
(4) both a lipid-lowering medication and RAS blockade.

### Primary Outcome

The primary outcome was adherence to both lipid-lowering medications (including statins and fibrates) and RAS blockade [Angiotensin Converting Enzyme inhibitor (ACEi) or Angiotensin Receptor Blocker (ARB)] at 12 months post-AMI. Lipid-lowering and RAS blockade treatments were selected to examine guideline-indicated medications because both medication classes in Australia require a prescription and so are systematically captured in prescription claims data. In contrast, antiplatelet medications, which are also recommended for secondary prevention, were not included because a large number are available without a prescription and so purchasing patterns using routinely collected data are unreliable (Australian Bureau of Statistics, 1011; Britt et al., 2015).

Prescriptions filled were determined using the PBS Anatomical Therapeutic Chemical (ATC) Classification Level 5 codes. (WHO Collaboating Centre for Drug Statistics Methodology, 2021). The PBS records all claims dispensed under Australia’s universal public health insurance scheme that provides free or subsidized access to medications. The codes used for lipid-lowering medications were C10AA/AB/BA/BX, and for RAS blockade were C09AA/BA/CA/DA (Supplementary Appendix Supplementary Table S3).

### Calculating Proportion of Days Covered

A participant was considered adherent to medication if they had access to the medication at least 80% of the time. Electronic dispensing data were used to identify the date of supply of a medication and the quantity supplied and hence to calculate the proportion of days covered (PDC) by these purchases.

Further details and assumptions (Arnet et al., 2016) for the calculation of PDC can be found in the supplementary material. Dual therapy adherence is addressed by requiring that participants are in receipt of both medication classes at the same time over the time interval of interest (Supplementary Appendix Supplementary Figure S2).

### Statistical Analyses

The analysis was performed in three parts: 1) variable selection was performed to subset the large number of variables using clinical relevance and boosted regression tree (BRT) models; 2) adherence over time was observed, stratified by key variables selected in 1; and 3) multivariable regression for the primary outcome was performed on the subset of variables selected in 1.

### Boosted Regression Trees for Variable Selection

The BRT (Elith et al., 2006; Elith et al., 2008) approach to variable selection allows a subset of variables to be identified according to their relative influence in explaining the variability of the outcome and frees analysis from the constraints of variable selection via p-value-based algorithms. (Derksen and Keselman, 1992; Thompson, 1995; Friedman et al., 2000; Jackson, 2008; Wasserstein and Lazar, 2016; Smith, 2018).

### Adherence Over Time

The proportion of individuals who were adherent over time was also assessed, both for the overall cohort and stratified by variables found to be influential in the BRT analysis. PDC was assessed in quarterly intervals from 12 months prior to the AMI event until the end of follow up. Assessing adherence prior to the AMI allowed the impact of the AMI event on medication use to be examined. Proportions are displayed with 95% binomial confidence intervals. Longitudinal adherence to the individual medication classes was also assessed, using the previously defined lipid-lowering medication and RAS blockade ATC codes.

### Multivariable Regression

Multivariable logistic regression was used to model the association of adherence with the proportion of individuals with a PDC ≥80% at the primary outcome period (between 9 and 12 months post-AMI). Variables included in the model were those informed by the BRT analysis as well as those considered to be of clinical importance based on prior literature and expert clinician input. Some highly correlated variables (such as hyperlipidemia) were removed for the primary analyses but were included in sensitivity analyses. Categorical exposure variables were modelled using linear terms and their effects illustrated using forest plots of the odds ratios and 95% confidence intervals. Age was categorized into 10-years age groups. A sensitivity analysis was performed with additional variables including seven comorbidities and highly correlated variables previously removed.

The analysis was performed using SAS version 9.4, SAS Enterprise Guide 7.1 and R version 3.6.2 (R Core Team, 2014). Cohort identification, adherence calculations and manipulations of APDC, MBS and PBS data were completed in SAS. R was used for boosted regression tree (gbm 2.1.5 (Greenwell et al., 2020)), generalized additive models (Wood, 2017) (mgcv 1.8–28), further logistic regression and statistical graphics (ggplot2 (Wickham, 2016), visreg (Breheny and Burchett, 2017)).

### Patient and Public Involvement

Participant recruitment and surveying were performed by the Sax Institute as part of the 45 and Up Study. Results from this research will be disseminated to the community through The George Institute’s social media platforms and website, and directly to the 45 and Up Study participants via established Sax Institute channels.

## Results

In total, 14,200 individuals were identified as surviving an index AMI between 2006 and 2014 and meeting the eligibility criteria (Supplementary Appendix Supplementary Figure S2). The mean age was 69.9 years at AMI (SD = 10.45) with 38.7% being female, and median follow-up time of almost 4 years (44.5 months IQR: 43.7 months). Key demographic, cardiovascular risk factors, comorbidities and AMI event characteristics are shown in Table 1.

**TABLE 1:** Cohort characteristics of people with a first AMI meeting eligibility requirements by prior exposure.

Characteristics	Treatment naïve (N = 4,011)	Prior lipid lowering exposure (N = 3,768)	Prior RAS blockade exposure (N = 1,729)	Prior lipid lowering and RAS blockade exposure (N = 4,692)	Complete eligible cohort (N = 14,200)
**Demographic**					
**Sex (Female)**	1,636 (40.8%)	1,260 (33.4%)	796 (46.0%)	1,804 (38.4%)	5,496 (38.7%)
**Comorbidities**					
Hypertension	1,544 (38.5%)	1,750 (46.4%)	1,476 (85.4%)	4,217 (89.9%)	8,987 (63.3%)
Hyperlipidaemia	910 (22.7%)	2,445 (64.9%)	367 (21.2%)	3,735 (79.6%)	7,457 (52.5%)
Type 2 Diabetes	354 (8.8%)	596 (15.8%)	276 (16.0%)	1,523 (32.5%)	2,749 (19.4%)
Chronic kidney disease	458 (11.4%)	442 (11.7%)	363 (21.0%)	1,031 (22.0%)	2,294 (16.2%)
Cancer	1,643 (41.0%)	1,474 (39.1%)	805 (46.6%)	2,130 (45.4%)	6,052 (42.6%)
Depression	541 (13.5%)	462 (12.3%)	198 (11.5%)	575 (12.3%)	1,776 (12.5%)
Stroke	145 (3.6%)	154 (4.1%)	92 (5.3%)	381 (8.1%)	772 (5.4%)
**Characteristics of AMI**					
Mean age at AMI (SD)	67.5 (11.73)	67.1 (9.94)	73.6 (9.49)	72.8 (8.71)	69.9 (10.45)
Median length of stay (Q1; Q3)	2.0 (1.0; 6.0)	1.0 (1.0; 5.0)	3.0 (1.0; 8.0)	2.0 (1.0; 7.0)	2.0 (1.0; 6.0)
**STEMI/Non-STEMI**					
STEMI	380 (9.5%)	236 (6.3%)	144 (8.3%)	315 (6.7%)	1,075 (7.6%)
Non-STEMI	832 (21.0%)	567 (15.3%)	404 (23.7%)	918 (20.1%)	2,721 (19.5%)
Unspecified	2,799 (69.8%)	2,965 (78.7%)	1,181 (68.3%)	3,459 (73.7%)	10,404 (73.3%)
**Complications**					
Cardiac Arrest	26 (0.6%)	19 (0.5%)	12 (0.7%)	29 (0.6%)	86 (0.6%)
Cardiogenic Shock	11 (0.3%)	6 (0.2%)	8 (0.5%)	18 (0.4%)	43 (0.3%)
**Management strategy**					
Coronary angiogram only	469 (11.7%)	453 (12.0%)	189 (10.9%)	605 (12.9%)	1,716 (12.1%)
Percutaneous coronary intervention	2,930 (73.0%)	3,004 (79.7%)	1,205 (69.7%)	3,445 (73.4%)	10,584 (74.5%)
Coronary artery bypass grafting	135 (3.4%)	271 (7.2%)	74 (4.3%)	388 (8.3%)	868 (6.1%)
**Primary Care Engagement Prior to AMI**					
Primary care visits within 1 month^a^ (mean (SD))	0.9 (1.22)	1.0 (1.18)	1.2 (1.35)	1.2 (1.36)	1.1 (1.28)
Primary care visits between 2 and 6 months^b^ (mean (SD))	4.1 (4.00)	4.9 (3.97)	6.5 (5.15)	6.4 (4.56)	5.4 (4.46)
**Post-AMI**					
Primary care visits within 1 month^a^ (mean (SD))	1.4 (1.58)	1.5 (1.37)	1.9 (1.64)	1.8 (1.59)	1.6 (1.55)
Primary care visits between 2 and 6 months^b^ (mean (SD))	5.5 (5.32)	5.9 (4.77)	8.0 (5.89)	7.8 (5.58)	6.7 (5.46)

a GP, visits within 28 days of AMI.

b GP, visits between 29 and 180 days of AMI.

Overall adherence increased from 18.2% (95% CI: 17.4–18.6%) at the time of the AMI to 28.0% (95% CI: 27.3–28.8%) within the first 3 months post AMI. Adherence increased to 29.5% (95% CI: 28.8–30.0%) by 12 months. After 12 months the overall adherence was maintained. By 24 months post AMI overall adherence was 28.9% (95% CI: 28.1–29.8%) (Figure 2 grey downward vertex triangle).

### Variable Selection

**FIGURE 1:**
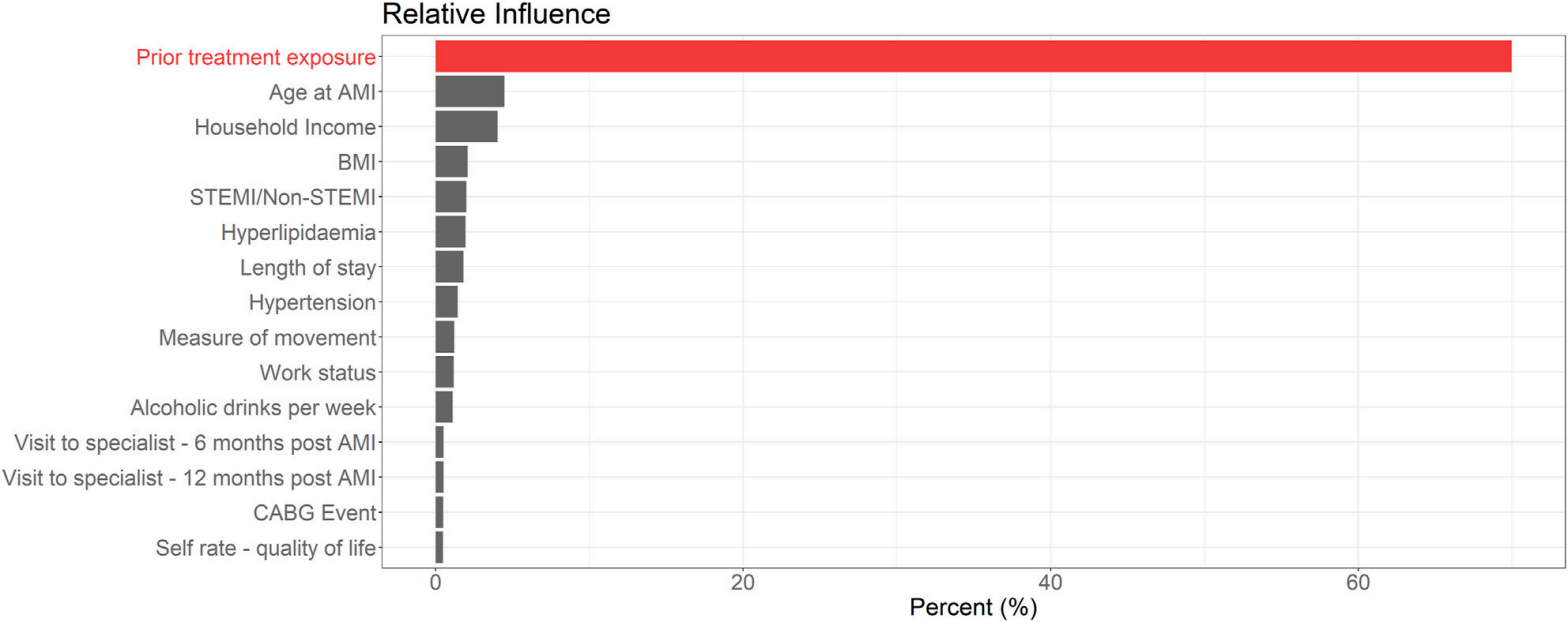
Top 15 variables when ranked *via* relative influence from boosted regression tree models for medication adherence 12 months post AMI.

Of 51 characteristics included in the BRT, the 15 variables with the highest relative influence are shown in Figure 1. Prior treatment exposure was the most influential variable by a considerable margin, minimizing the loss function in over 70% of BRT models. All other variables had a relatively minor influence (Supplementary Appendix Supplementary Figure S3).

### Analyses by Prior Treatment Exposure

In total 28.2% (n = 4,011) of the study cohort were treatment naïve, 26.5% (n = 3,768) had been previously exposed to lipid-lowering medication only, 12.2% (n = 1,729) to RAS blockade only, and 33.0% (n = 4,692) had been exposed to both a lipid-lowering medication and RAS blockade.

The main differences in characteristics by prior treatment exposure group relate to higher rates of pre-AMI diagnoses of hyperlipidemia and hypertension and higher primary care utilization both before and after the AMI in the groups with prior RAS blockade use and those with lipid-lowering and RAS blockade medication use compared to the other two groups (Table 1). Further cohort characteristics are in the supplementary material (Supplementary Appendix Supplementary Table S4).

**FIGURE 2:**
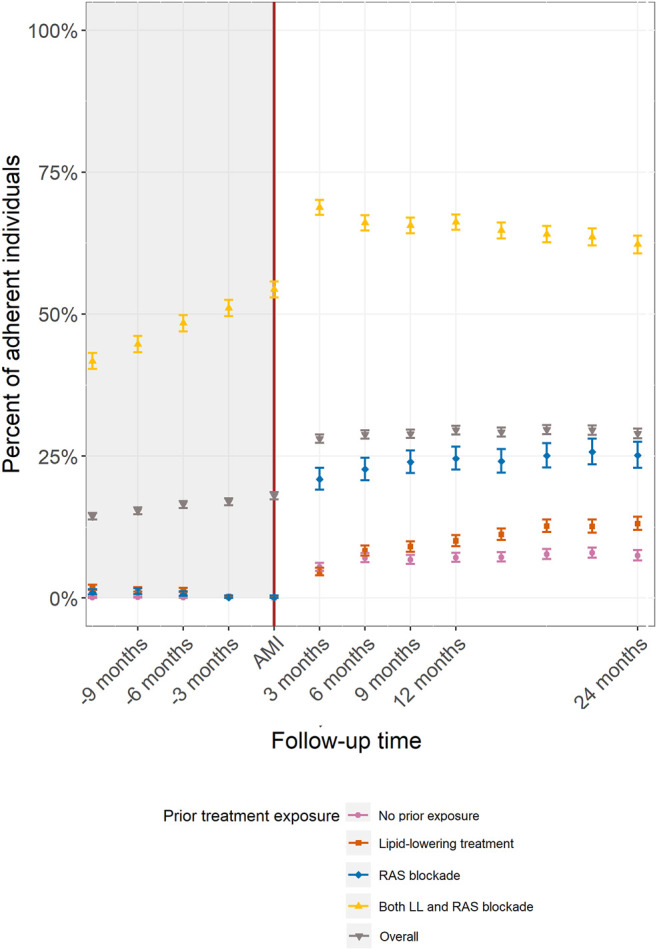
Adherence to both lipid-lowering and RAS blockade treatments by pre-AMI treatment exposure.

The trend in post-AMI adherence differed according to pre-AMI treatment exposure (Figure 1). In people previously exposed to both medications, the proportion of individuals with a PDC ≥80% slowly increased in the 12 months prior to the AMI event, with 54.3% (95% CI: 52.9–55.8%) having a PDC ≥80% at the time of the event. Adherence rates then increased between the AMI and 3 months post-AMI to 68.8% (95% CI: 67.5–70.1%) and plateaued to 66.2% by 12 months (95% CI: 64.8–67.5%). The three other groups defined by prior exposure showed a moderate increase in adherence following the AMI with minimal change thereafter. (Figure 2).

### Multivariable Analyses of Adherence

**FIGURE 3:**
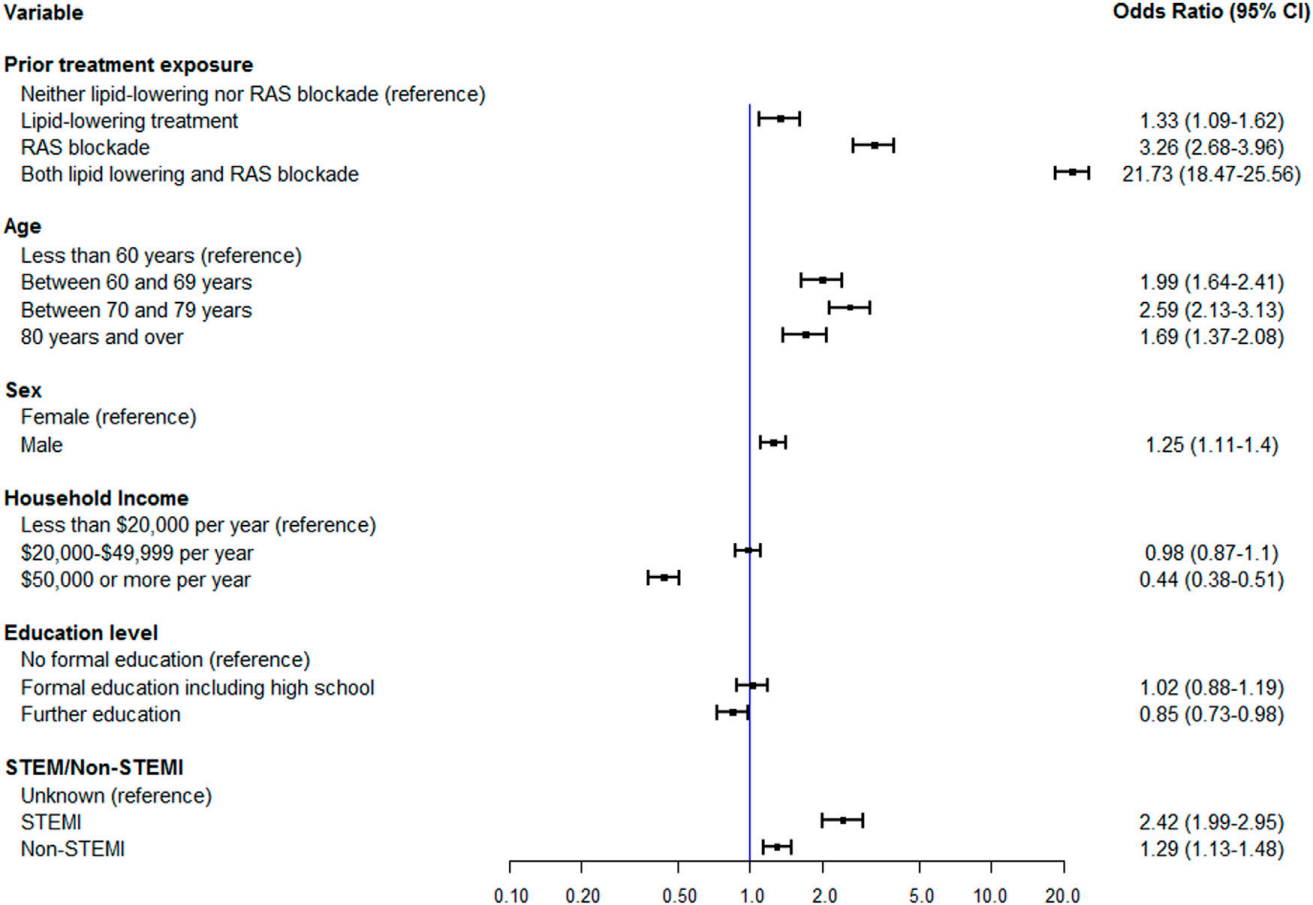
Multivariable logistic regression of adherence at 12 months post AMI by prior treatment exposure including age, sex, income, education level and STEMI/Non-STEMI status.

After adjustment for the most influential variables in the BRT analysis (age, income and AMI severity) and clinically informed variables (sex and education level), post-AMI medication adherence was clearly different in groups defined by prior treatment exposure (Figure 3).

Compared to treatment naïve, those who had been dispensed both medication classes in the 12 months prior to their AMI were over 9 times more likely (RR = 9.3, 95% CI: 8.54–10.13) to be adherent to both medication classes following the index AMI with an odd ratio of 21.73 (95% CI: 18.47 to 25.56, n = 4,692). Those exposed to only lipid-lowering medication were 33% more likely to be adherent (OR 1.33, 95% CI: 1.09 to 1.62, n = 3,768) and those recently treated with only RAS blockade had a 3-fold increase (OR 3.26, 95% CI: 2.68 to 3.96, n = 1729) than treatment naïve. Prior treatment exposure explained 31.3% of the variance in adherence associated with the adherence in the outcome interval in the overall cohort (R2_adjusted_ = 0.353).

Results were similar in a sensitivity analysis performed on an expanded variable set which included prior treatment exposure, age, sex, income, education level, AMI severity and seven comorbidities. In these analyses, the odds ratio for people previously dispensed both medications compared with no prior treatment exposure was 14.49 (95% CI: 12.14 to 17.29, n = 4,692) (Supplementary Appendix Supplementary Figure S4).

## Discussion

In this cohort study of 14,200 people hospitalized for a first AMI event, less than 30% of individuals were consistently adherent to both guideline-based medication classes at 12 months post-AMI. Adherence increased substantially in the 3-months interval following the AMI compared with utilization of the same medications prior to the AMI. However, the occurrence of the AMI itself only explained a minority of the post-infarct cardioprotective medication use. Prior medication use was the factor most strongly associated with adherence post-AMI. Nearly two-thirds of people taking these medicines pre-AMI were adherent at 12 months, and were around 9 times more likely to be adherent compared with those who had been dispensed neither classes prior to the AMI. This association was far stronger than other commonly-cited associations in the literature, including age and AMI event severity (Sabate, 2003; Heeley et al., 2010; Molfenter et al., 2012; Laba et al., 2013; Hall et al., 2016; Shang et al., 2019).

### Implications for Practice and Policy

The findings suggest that prescribing clinicians need robust systems in place to systematically determine prior medication exposure when assessing risks of non-adherence post-AMI. Intensified efforts are needed for all patients along with strategies that address both provider and patient barriers to adherence (Sabate, 2003; Kolandaivelu et al., 2014; Abbass et al., 2017; Burnier and Egan, 2019; Gast and Mathes, 2019). This applies to all patients but particularly for people with no prior use of lipid-lowering and RAS blockade medication. Only 7.1% of this group were optimally adherent to both therapies 12 months post-AMI. Prior treatment exposure is best identified via continuity of patient care. Policies around management plans supporting continuous relationships between patient and clinician should be encouraged, especially for patients with chronic or complex conditions.

Discharge counselling is an important component to patient health post-AMI. Our study shows that the first 3 months post-AMI have been shown to be crucial to developing good adherence. Guidelines exist around medication counselling that include patient education, medication management and disease management (Cai et al., 2013; Mathews et al., 2015a). The findings from this study indicate that the primary catalyst to adherence post event is not the initial AMI but previous medication exposure. Therefore, prior exposure needs to be a key consideration in the discharge medication counselling performed by the in-hospital pharmacist.

Integration of often fragmented health systems (World Health Organization, 2015) can support a wholistic and patient centered model of care which is an important component for medication adherence. New South Wales, Australian, has implemented a state-wide integrated care strategy. This strategy is focused on coordinating connection and communication between health care providers in the community and those in the hospital setting (NSW Health, 2020).

Community pharmacists can also play a critical role in enhancing support for patients at high risk of non-adherence. Although medication adherence interventions have had limited impact (Nieuwlaat et al., 2014), interventions have showed some success when administered through these services (Torres-Robles et al., 2021). In an environment of limited time and resources, targeting patients with the greatest need is key to making impacts in overall community adherence (Zullig et al., 2019). Prior treatment exposure is a tangible patient characteristic that a pharmacist can identify without a complex assessment. It is a scalable method to identify potential candidates for services that may help medication adherence in the initial months after an AMI including medication counselling, adherence support and follow-up (Jackevicius et al., 2008). Voluntary medication reviews by a pharmacist could be made available. Prior medication use should be a standard consideration in medication discussions.

### The Findings in the Context of Previous Evidence

The plateau in adherence overall and for all pre-event exposure groups at 12 months post-AMI differ from some (Jackevicius et al., 2002; Mathews et al., 2015b) but not all (Harrison et al., 2018) previous studies, which have mostly found adherence to decline over time. Differences between our study and previous reports, including adherence methodology, data sources and settings (comprehensive government-subsidized pharmaceutical benefits scheme vs. insurance claim data), making direct comparisons difficult.

The reason why prior medication utilization is associated with better post-infarct utilization cannot be determined from these results and may be mediated by multiple health service, practitioner and patient characteristics. Taking lifelong treatments is a complex adaptive process and it is possible patients who are already taking at least some of the recommended medication may not need to make as major changes in their medication-taking behavior post-AMI compared with those who were treatment naïve pre-event (Molfenter et al., 2012; Brown et al., 2016; Kini and Ho, 2018; Armitage et al., 2019).

It is reassuring that studies using single class adherence to lipid-lowering and RAS blockade medications from the United States (Akincigil et al., 2008) and Canada (Rasmussen et al., 2007) report higher levels of monotherapy adherence. Other single class studies using large data sources and advanced statistical techniques have also identified prior medication use as an important component to predict medication adherence when only a single class of medications is assessed (Zullig et al., 2019).

People who were exposed to both medications pre-AMI were older and have a greater burden of comorbidities, mainly hypertension and hyperlipidemia. The higher primary care utilization rates post-AMI observed in those on prior RAS blockade or simultaneous lipid-lowering medications and RAS blockade treatments may mean these two groups have a greater frequency of interactions with health care providers allowing more opportunity for renewal of prescriptions and appropriate adjustments to medication. Our study confirmed early reports that age (Chang et al., 2015) and AMI severity (Ge et al., 2019) are associated with treatment adherence. Similar to previous studies we found a non-linear associations of age with adherence in people aged 70 to 79-years-old higher than for younger or older age-groups (Chang et al., 2015).

We also observed that higher income was associated with lower adherence rates, when adjusted for education, age and AMI severity. There are varied associations between wealth and adherence in literature (Chernew et al., 2008; Abbass et al., 2017; González López-Valcárcel et al., 2017) and this may partly reflect variation in health system policies. As part of Australia’s universal health care coverage scheme, medications are heavily subsidized for low income individuals/households (e.g., a two or more person household with an income less than $50,000) and may contribute to the complex associations when assessing the relationship between wealth and adherence. Furthering the complex impact of income are financial threshold safety nets that are available to high health system users in the Australian community, for example people taking multiple medications or those with a high number of comorbidities. Eligible patients receive prescriptions and some health care services at a reduced cost. This scheme further removes cost barriers to medication and primary care visits for patients with high utilization patterns and multiple health needs.

### Strengths and Limitations of the Study

This study has many strengths from both the methodology and the data sets. BRT models identify key variables even in the presence of high correlation. In such cases the most informative variable will result in a higher relative influence score. The 45 and Up Study is large with over 250,000 participants with a range of variables including both survey and routinely collected data. Large and extensive data sets spanning such a diverse array of personal factors are uncommon. The adherence method used in this study is objective and comprehensive as it used dispensing data from the nationwide pharmacy network.

The use of national prescribing claims data enables complete follow-up of participants and is not prone to recall bias from self-report (Sabate, 2003; Garber et al., 2004). Through the utilization of medication dispensing data to identify exposure to medications prior to the AMI, associations with adherence following the AMI were able to be identified. Longitudinal studies of medication dispensing before and after a major event are not common.

A limitation is the inability to identify whether treatment gaps are due to non-prescribing by the care provider, or non-prescription filling or non-taking by the patient. In a recent study of general practice prescribing patterns in NSW, less than 60% of patients with an established diagnoses of cardiovascular disease diagnosis had a current prescription for guideline-recommended medications (Hespe et al., 2020). Another study limitation is that we lacked information on contraindications to the two medications. However, the highest rates of major contraindications to these medications is around 1.5–5% (mainly related to RAS blockade medications) (Bays, 2006; Caldeira et al., 2012; Clase et al., 2020) and therefore contraindications are unlikely to explain the overall low adherence rates observed in this study (Keen et al., 2014; Laufs et al., 2015; Ward et al., 2019). The data used in this study extends from 2006 to 2014. Nevertheless, the nature of medication adherence and the influencers of behavior are unlikely to have changed substantially in the 8 years since 2014. Our conclusions therefore remain applicable. Guideline-recommended cardioprotective medications were adjusted to reflect best practice at the time the data was collected. Finally, the results relate to people who have experience an initial AMI and may not be generalizable to those experiencing multiple events.

Large and constantly evolving data sources offer ready opportunities for further research. In the context of this study, examining the influence of poly pills (Roshandel et al., 2019) on medication adherence could highlight an interesting influence with both the ease of one pill and the mitigated side effect from the dual treatment. Further, the scope of personal characteristics can be further examined with greater interrogation into hereditary conditions and comorbidities such as post AMI mental health and how this impacts medication adherence (Thombs et al., 2006). As follow up progresses data will yield a greater number of subsequent cardiovascular events. Adherence in relation to subsequent AMIs could also be examined.

## Conclusion

Sub-optimal adherence to best practice care guidelines is a complex and intractable challenge in many areas of health care. Although a robust evidence base for secondary prevention of cardiovascular disease events for people who have had an AMI exists, a large proportion of people are not receiving the benefits of pharmacotherapy support. This adherence gaps contributes to avoidable personal burden and societal costs. Overall the optimal use of life-saving, low cost therapies after an AMI is low. These low adherence rates indicate that systematic appraisal of the risk of non-adherence in the immediate post-AMI period represents a potential opportunity to improve outcomes for individuals. Particular attention should be paid to a patient’s prior cardiovascular treatments pre-AMI. Results from this large data analysis show that prior treatment is a key influence to post AMI medication adherence. Of crucial concern are people who have had no prior experience with taking the recommended medications.

## Data Availability

The data analyzed in this study was obtained from https://www.saxinstitute.org.au/our-work/45-up-study/, the following licenses/restrictions apply: institutional restrictions. Requests to access these datasets should be directed to the Sax Institute, 45andUp.research@saxinstitute.org.au.
