# Supplementary material for: Prior Cardiovascular Treatments—A Key Characteristic in Determining Medication Adherence After an Acute Myocardial Infarction
Source: Front Pharmacol. 2022 Mar 7;13:834898. doi: 10.3389/fphar.2022.834898 (PMC8940291; doi:10.3389/fphar.2022.834898)
Supplement: Supplementary file 1 [file DataSheet1.docx]

Supplementary Material

# Supplementary Methods

## The 45 and Up Study

Between 2006 and 2009, invited participants were asked to complete a postal questionnaire on healthy ageing and consent to ongoing linkage to their data held in routinely collected databases. The 45 and Up Study had an 18% response rate covering approximately 11% of the NSW population aged 45 years and over(1) and has been shown to report near representative estimates for many of the various measures estimated by the NSW health survey(2). In the EXTEND45 Study, 45 and Up Study participants and their baseline questionnaire responses have been linked to routinely collected administrative health datasets, outpatient laboratory results from laboratory service providers, and the Australia and New Zealand Dialysis and Transplant (ANZDATA) registry.

## Study Cohort

AMI diagnoses were identified using standardised codes from the International Statistical Classification of Disease and Related Health Problems, Tenth Revision, Australian Modification (ICD-10-AM) codes (Table S1)(3).

## Table S1: ICD-10 AM code for a diagnosis of acute myocardial infarction

| ICD 10-AM Code | Description |
| --- | --- |
| I21 | Acute myocardial infarction |
| I21.0 | Acute transmural myocardial infarction of anterior wall |
| I21.1 | Acute transmural myocardial infarction of inferior wall |
| I21.2 | Acute transmural myocardial infarction of other sites |
| I21.3 | Acute transmural myocardial infarction of unspecified site |
| I21.4 | Acute sub-endocardial myocardial infarction |
| I21.9 | Acute myocardial infarction, unspecified |
| I25.0 | Atherosclerotic cardiovascular disease so described |
| I25.1 | Atherosclerotic heart disease |
| I25.10 | Atherosclerotic heart disease of unspecified vessel |
| I25.11 | Atherosclerotic heart disease of native coronary artery |

### Further details regarding selection criteria and Index AMI identification

Hospitalisation records and self-reported results were used to validate an incident AMI. If either data source indicated a prior event the patient was excluded. We also excluded participants who had undergone a coronary revascularisation procedure during 2005 (the 12-month period prior to 45 and Up Study enrolment), or who answered positively to the 45 and Up Study baseline questionnaire “Q*25. In the last month have you been treated for heart attack or angina?”*

## Covariates

Demographic, socioeconomic, lifestyle and clinical characteristics were derived from self-reported information from the 45 and Up Study baseline questionnaire and included: sex, age, highest educational qualification, relationship status, annual household income, smoking status, alcohol consumption, body mass index (BMI, in kg/m^2^). Pre-existing co-morbid conditions were based on a combination of self-report, medication use, hospital admission and laboratory data, where available. Remoteness of residence was derived based on participants’ residential postcode using the Australian Bureau of Statistics’ Accessibility/Remoteness Index of Australia (ARIA+).(4) Health service use was measured by the number of primary health care physicians and medical specialist visits identified in the MBS database and was used as a marker for engagement with the health system. AMI characteristics were obtained from the APDC dataset, using ICD-10-AM and Australian Classification of Health Interventions codes. An AMI was characterised as either a ST-elevation myocardial infarction (STEMI), non-STEMI or unspecified. Procedures such as percutaneous coronary intervention (PCI), coronary artery bypass grafting (CABG) or a coronary angiography during the AMI admission were also recorded (Table S2).

## Table S2: Demographics, pre-existing co-morbid diagnoses and service utilisation was based on a combination of self-report, medication use, hospital admission, pathology data and MBS data.

| Characteristics | Source | Details |
| --- | --- | --- |
| Sex | 45 & Up | Obtained from the Services Australia database, participants were provided a gender-specific questionnaire. |
| Relationship status | 45 & Up | Q14. What best describes your current situation? Answers: single; married; de facto/ living with partner; widowed; divorced; separated. |
| Highest education qualifications | 45 & Up | Q5. What is the highest qualification you have completed? |
| Health insurance status | 45 & Up | Q50. Besides Medicare, what form of health insurance do you have? (5 options) |
| Working status | 45 & Up | Q47. What is your current work status? |
| Household income | 45 & Up | Q46. What is your usual yearly HOUSEHOLD income before tax, from all sources? (Please include benefits, pensions, superannuation, etc) Answers: less than $5,000 per year; $5,000-$9,999 per year; $10,000-$19,999 per year; $20,000-$29,999 per year; $30,000-$39,999 per year; $40,000-$49,999 per year; $50,000-$69,999 per year; $70,000 or more per year or I would rather not answer this question. |
| Smoking status | 45 & Up | Q11. Have you ever been a regular smoker? Answers: Yes or No. If yes: How old were you when you started smoking regularly? (age in years); Are you a regular smoker now? Answers: Yes or No; About how much do you/ did you smoke on average each day? (If you are an ex-smoker, how much did you smoke on average when you smoked?) Answer in cigarettes per day or pipes and cigars per day. |
| BMI (kg/m2) | 45 & Up | Q3. How tall are you without shoes? AND Q4. About how much do you weigh? |
| Alcohol history | 45 & Up | Q12. About how many alcoholic drinks do you have each week? (One drink = glass of wine, middy of beer or nip of spirits, put 0 if you do not drink or have less than one drink each week) |
| Hypertension | 45 & Up | Q24. Has a doctor ever told you that you have high blood pressure?  OR  Q25 In the last month have you been treated for high blood pressure? |
| Hyperlipidaemia | 45 & Up | Q23 Have you taken Lipitor for most of the last 4 weeks?  OR  Have you taken Pravachol for most of the last 4 weeks?  Or Have you taken Zocor, Lipex for most of the last 4 weeks?  OR  Q25 In the last month have you been treated for high blood cholesterol? |
| diabetes (Type 1 or 2) | 45 & Up / Pathology / PBS | Q24. Has a doctor ever told you that you have diabetes? OR  dispensed at least one glucose-lowering medication or insulin analogue based on PBS data OR  a pathology record of at least one HbA1c result > 6.5%,  OR  a pathology record of a plasma glucose >11.1 mmol/L, taken as part of an oral glucose tolerance test  OR a pathology record of a fasting plasma glucose >7.0 mmol/L |
| Chronic kidney disease | Pathology | an estimated glomerular filtration rate (eGFR) value <60 mL/min/1.73 m^2^ at any time before the index AMI |
| Cancer | 45 & Up | Q24. Has a doctor ever told you that you have cancer? |
| Depression | 45 & Up | Q24. Has a doctor ever told you that you have depression? |
| Stroke | ICD 10-AM Code | I61.0- I61.6, I61.8, I61.9, I62.0, I62.1, I62.9, I63.0-I63.6, I63.8, I63.9, I64, I69.0-I69.4, I69.8 |
| AMI Severity | ICD 10-AM Code | STEMI: I21.0-3 Non-STEMI: I21.4 Unspecified STEMI: I21.9 |
| Type of AMI | **-** |  |
| AMI management strategy | ACHI code block numbers | PCI: 671 CABG: 672-679 Coronary Angiography: 670 |
| GP use | MBS item numbers | 3 4 20 23 24 35-37 43 44 47 51 |

## Primary outcome

Prescriptions filled were determined using the PBS Anatomical Therapeutic Chemical (ATC) Classification Level 5 codes.(5) The PBS records all claims dispensed under Australia’s universal public health insurance scheme that provides free or subsidised access to medications. The codes used for lipid-lowering medications were C10AA/AB/BA/BX, and for RAS blockade were C09AA/BA/CA/DA (Table S3)

## Table S3: ATC level 5 codes used to identify a RAS blockade and Lipid lowering medications in the PBS

| Drug class | ATC Level 5 codes |
| --- | --- |
| Lipid lowering | C10AA01 C10AA03-C10AA05 C10AA07 C10AB04 C10AB05 C10BA02 C10BA05 C10BX03 |
| RAS blockade | C09AA01-C09AA06 C09AA09 C09AA10 C09BA02 C09BA04 C09BA06 C09BA09 C09CA01-C09CA04 C09CA06-C09CA08 C09DA02-C09DA04 C09DA06-C09DA08 |

### Calculating PDC

A participant was considered adherent to medication if they had access to the medication at least 80% of the time.

Electronic dispensing data were used to identify the date of supply of a medication and the quantity supplied and hence to calculate the proportion of days covered (PDC) by these purchases. Hospital records were used to identify date of discharge and subsequent hospitalisations within the follow-up period. Date of censoring was identified via hospitalisation records and RBDM data. Adherence was measured over 3-month intervals (Figure S1). An assumption of seven days of post-discharge medication supply was made for the first interval following discharge, accounting conservatively for the typical NSW hospital practice of supplying a few days medication on discharge.

### Figure S2: Steps associated with the calculation of adherence


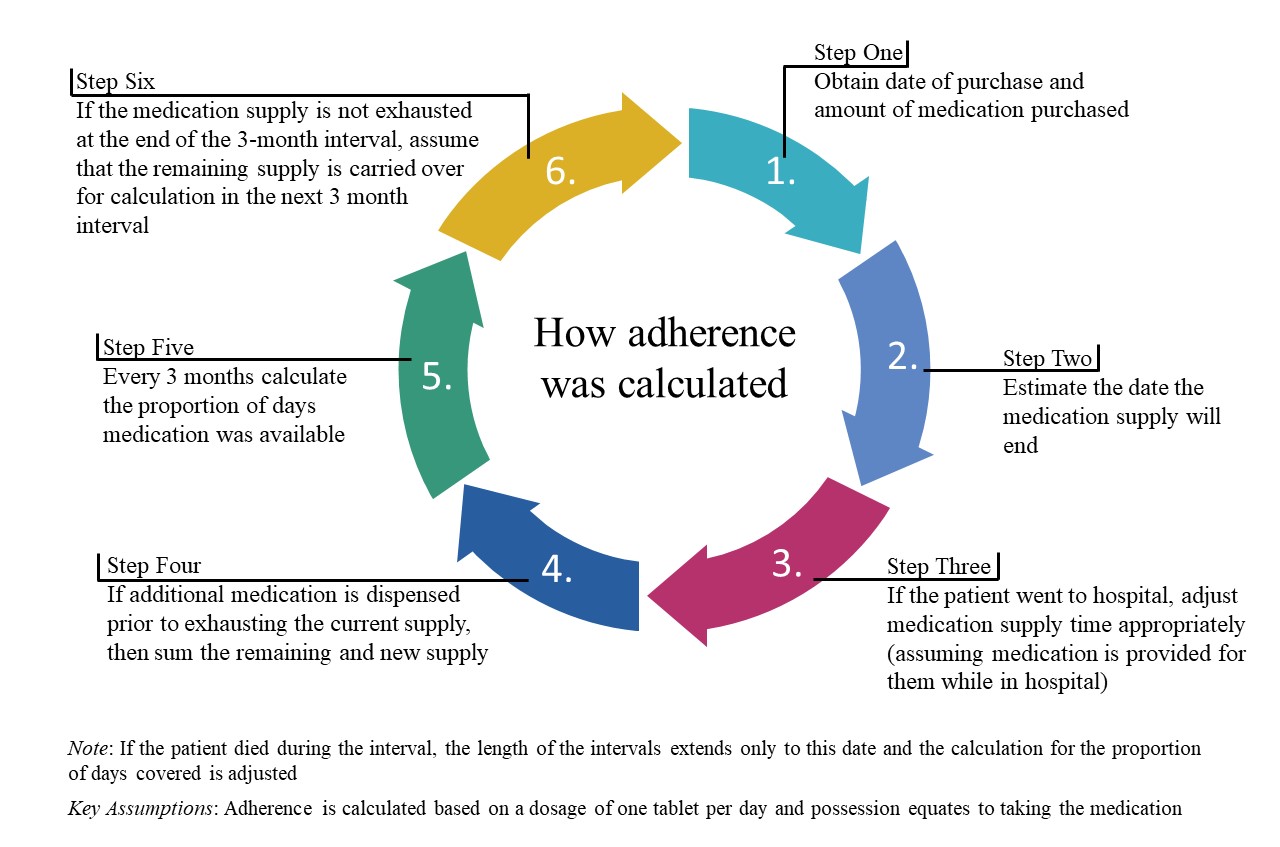


Further assumptions for the calculation of PDC include:

- Dispensing records being complete, comprehensive and accurate
- The day on which the dispensed medication was first taken (e.g. on day of purchase or after the end of a previous supply)
- The first medicine was taken the day the medication was purchased or, in instances where a prescription was filled before the end of a previous packet, the first medicine was taken the day immediately after the end of supply.
- Medication was taken based on one pill per day dosage (based on clinical knowledge of medications of interest)
- Medications were not purchased or obtained from another person or venue (e.g. hospital pharmacy)
- No unknown treatment interruptions or dosing change occurred during the observation period, the only exception being if a patient was admitted to hospital during the observation period
- Possession of the medication was equated with taking the medication.

Calculation of the PDC involves using dispensing data to establish the number of dosages (assumed to be equivalent to number of pills) available in a packet and assess the proportion of days an individual had access to the medication over an interval. Careful calculation of PDC takes into consideration refilling of supplies, generic or therapeutic substitutions, oversupply of medication as well as alterations in the length of the observation interval due to non-exposure periods or censoring. Dual therapy adherence is addressed by requiring that participants are in receipt of both medication classes at the same time over the time interval of interest (Figure S1).

## Figure S2: Requirements for dual adherence


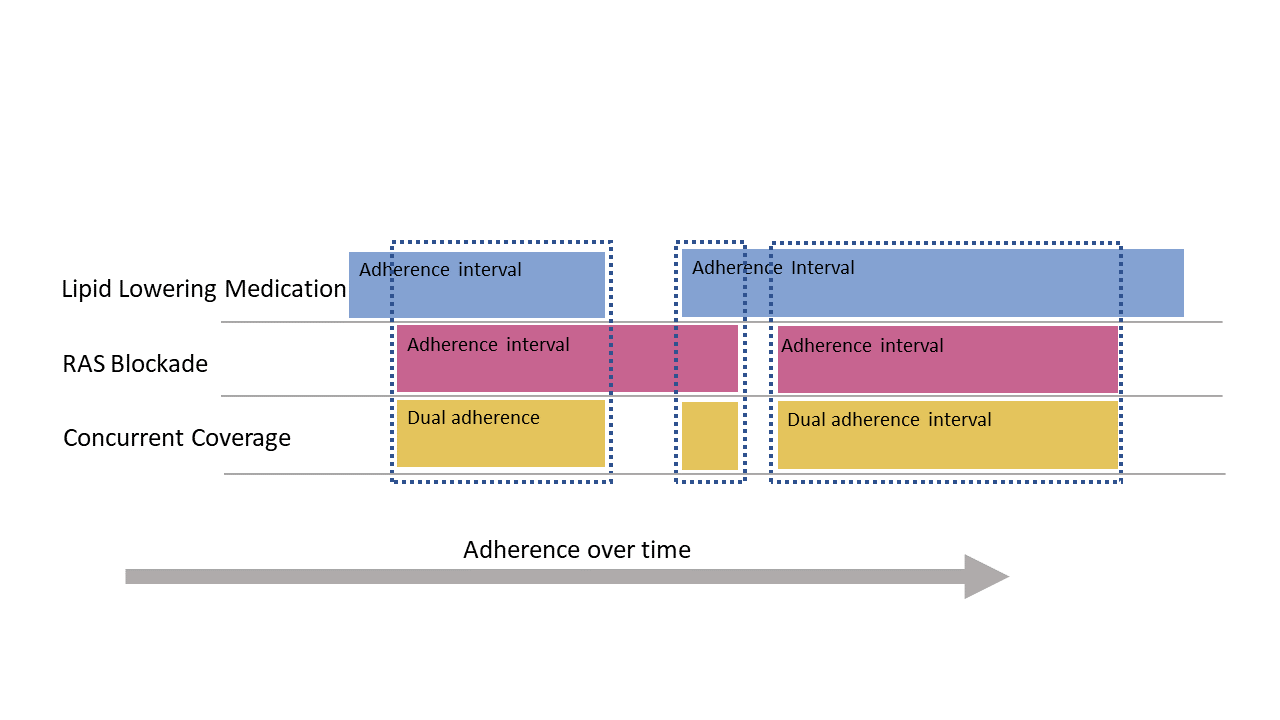


### Boosted Regression Trees for variable selection

The BRT(6, 7) approach to variable selection allows a subset of variables to be identified according to their relative influence in explaining the variability of the outcome and frees analysis from the constraints of variable selection via p-value-based algorithms.(8-11) The approach uses boosting algorithms to iteratively fit weighted regressions trees. The greedy BRT algorithm adjusts for poorly fitting observations with each iteration of the algorithm and boosting set. Regression trees (often exceeding 1000) are combined to create an ensemble learning measure. Through this measure, each variable is provided a relative influence which is a measure of relative importance. A relative influence score is measured between 0 and 100% and is based on both the percentage reduction in the loss function and the boosting set(12). Once ranked, these scores can be used to identify highly informative variables. Variables with large relative importance scores can be interpreted as being highly independently associated with adherence.

# Additional Results

# Figure S2: Eligibility Criteria Flow Diagram
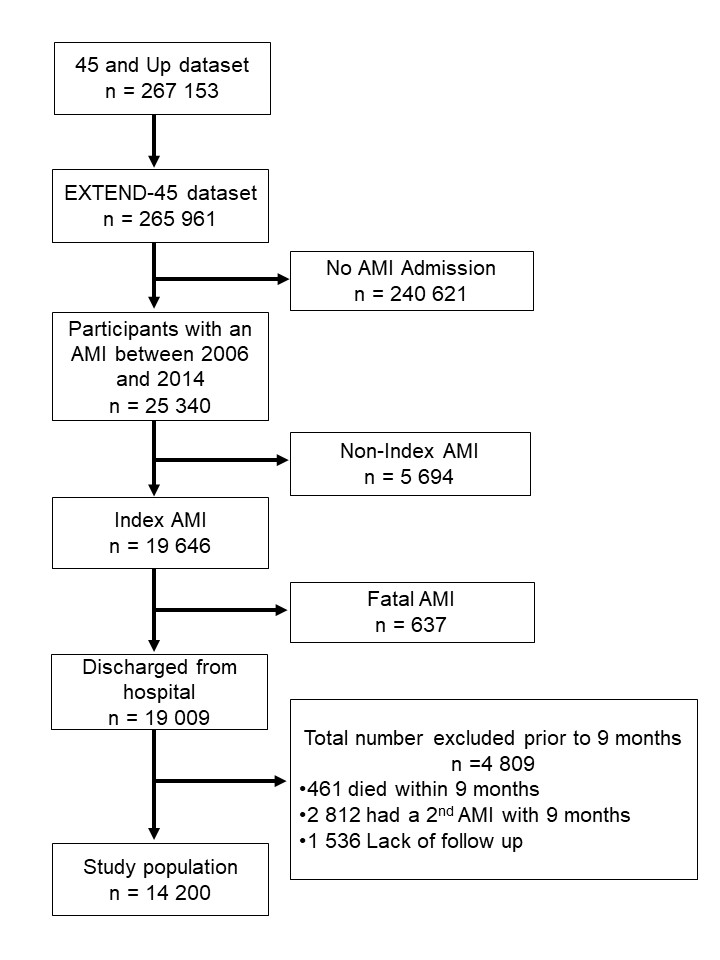


## Figure S3: Variables ranked by relative influence variables for 12-month adherence from the boosted regression tree models


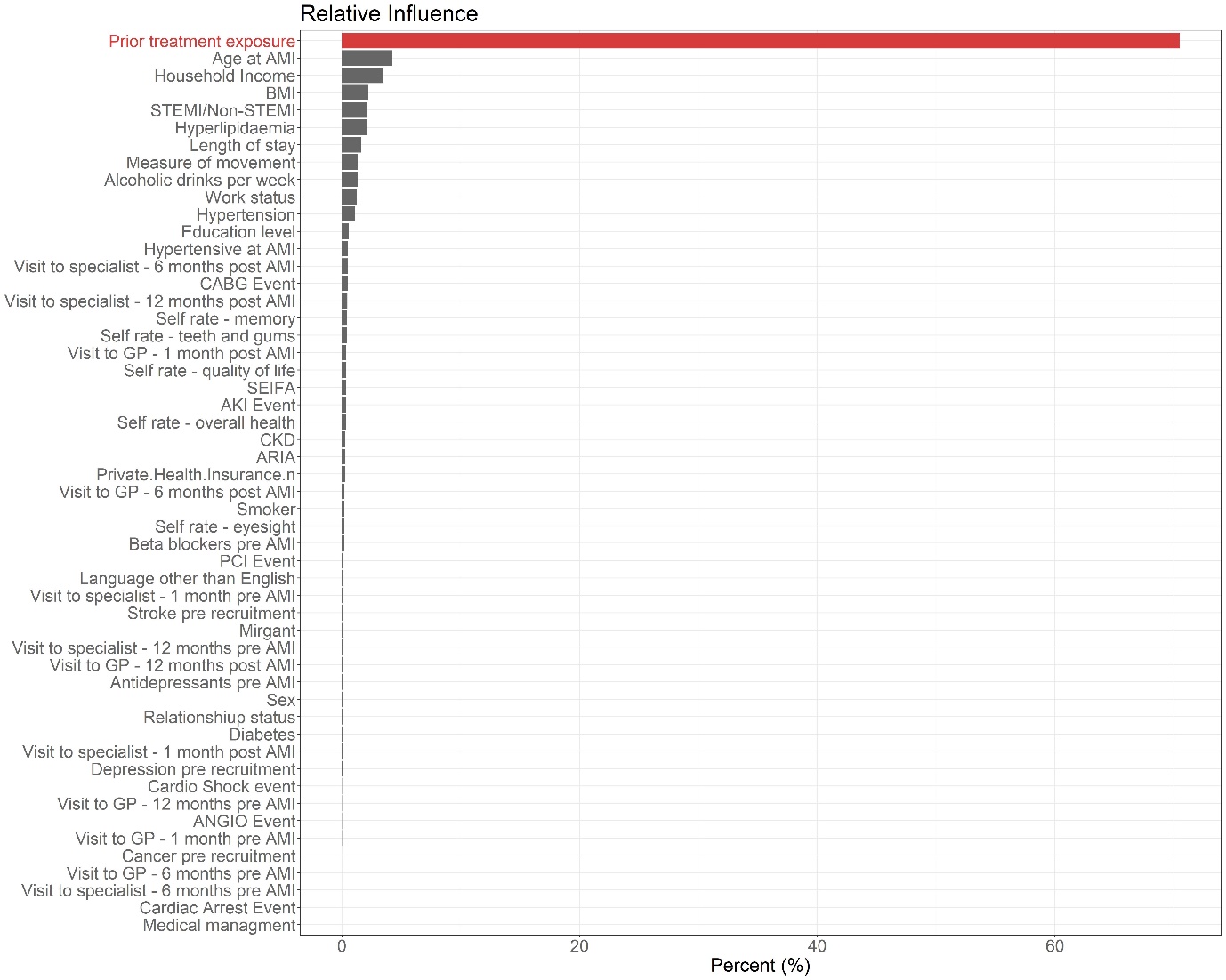


## Table S4: Further results for the characteristics of cohort by pre-AMI treatment exposure

| **Characteristics** | **No Prior treatment (N=4011)** | **Prior lipid lowering exposure (N=3768)** | **Prior RAS blockade exposure (N=1729)** | **Prior exposure to both LL and RAS (N=4692)** | **Complete eligible cohort (N=14200)** |
| --- | --- | --- | --- | --- | --- |
| **Demographic** |  |  |  |  |  |
| **Sex (Female)** | 1636/4011 (40.8%) | 1260/3768 (33.4%) | 796/1729 (46.0%) | 1804/4692 (38.4%) | 5496/14200 (38.7%) |
| **Relationship Status** |  |  |  |  |  |
| In relationship | 2930/3977 (73.7%) | 2979/3747 (79.5%) | 1194/1719 (69.5%) | 3467/4666 (74.3%) | 10570/14109 (74.9%) |
| **Highest educational qualifications** |  |  |  |  |  |
| Secondary school or below | 1670/3914 (42.7%) | 1569/3700 (42.4%) | 917/1684 (54.5%) | 2509/4586 (54.7%) | 6665/13884 (48.0%) |
| Trade or diploma | 1380/3914 (35.3%) | 1272/3700 (34.4%) | 527/1684 (31.3%) | 1460/4586 (31.8%) | 4639/13884 (33.4%) |
| Tertiary education or above | 864/3914 (22.1%) | 859/3700 (23.2%) | 240/1684 (14.3%) | 617/4586 (13.5%) | 2580/13884 (18.6%) |
| **Health insurance status^a^** |  |  |  |  |  |
| Universal health care alone | 2043/4011 (50.9%) | 2255/3768 (59.8%) | 736/1729 (42.6%) | 2134/4692 (45.5%) | 4848/14200 (34.1%) |
| Universal health care plus additional hospital cover | 513/4011 (12.8%) | 562/3768 (14.9%) | 275/1729 (15.9%) | 834/4692 (17.8%) | 2184/14200 (15.4%) |
| Universal health care, additional hospital and out of hospital cover | 1455/4011 (36.3%) | 951/3768 (25.2%) | 718/1729 (41.5%) | 1724/4692 (36.7%) | 7168/14200 (50.5%) |
| **Working status^b^** |  |  |  |  |  |
| Not working (including fully retired) | 2163/3947 (54.8%) | 1993/3725 (53.5%) | 1399/1703 (82.1%) | 3811/4642 (82.1%) | 9366/14017 (66.8%) |
| Part time | 648/3947 (16.4%) | 602/3725 (16.2%) | 164/1703 (9.6%) | 435/4642 (9.4%) | 1849/14017 (13.2%) |
| Full time | 1136/3947 (28.8%) | 1130/3725 (30.3%) | 140/1703 (8.2%) | 396/4642 (8.5%) | 2802/14017 (20.0%) |
| **Household income** |  |  |  |  |  |
| <$20,000 | 712/3774 (18.9%) | 643/3600 (17.9%) | 557/1578 (35.3%) | 1471/4341 (33.9%) | 3383/13293 (25.4%) |
| $20,000 - $49,999 | 997/3774 (26.4%) | 924/3600 (25.7%) | 495/1578 (31.4%) | 1353/4341 (31.2%) | 3769/13293 (28.4%) |
| $50,000 - $69,999 | 1374/3774 (36.4%) | 1405/3600 (39.0%) | 205/1578 (13.0%) | 643/4341 (14.8%) | 3627/13293 (27.3%) |
| $70,000 or more | 691/3774 (18.3%) | 628/3600 (17.4%) | 321/1578 (20.3%) | 874/4341 (20.1%) | 2514/13293 (18.9%) |
| **Cardiovascular risk factors** |  |  |  |  |  |
| **Smoking Status** |  |  |  |  |  |
| Current smoker | 333/3995 (8.3%) | 257/3755 (6.8%) | 88/1725 (5.1%) | 234/4676 (5.0%) | 912/ 14151 (6.4%) |
| Previous smoker | 1646/3995 (41.2%) | 1614/3755 (43.0%) | 727/1725 (42.1%) | 2133/4676 (45.6%) | 6120/ 14151 (43.2%) |
| Never smoker | 2016/3995 (50.5%) | 1884/3755 (50.2%) | 910/1725 (52.8%) | 2309/4676 (49.4%) | 7119/ 14151 (50.3%) |
| **BMI (kg/m^2^)** |  |  |  |  |  |
| <18.5 | 70/3707 (1.9%) | 27/3527 (0.8%) | 24/1573 (1.5%) | 31/4310 (0.7%) | 152/ 13117 (1.2%) |
| 18.5-<25.0 | 1268/3707 (34.2%) | 1075/3527 (30.5%) | 483/1573 (30.7%) | 1086/4310 (25.2%) | 3912/ 13117 (29.8%) |
| 25-<30 | 1537/3707 (41.5%) | 1549/3527 (43.9%) | 634/1573 (40.3%) | 1780/4310 (41.3%) | 5500/ 13117 (41.9%) |
| 30+ | 832/3707 (22.4%) | 876/3527 (24.8%) | 432/1573 (27.5%) | 1413/4310 (32.8%) | 3553/ 13117 (27.1%) |
| **Alcohol history (drinks per week)** |  |  |  |  |  |
| 0 to 6 drinks | 2354/3900 (60.4%) | 2186/3696 (59.1%) | 1084/1679 (64.6%) | 2843/4552 (62.5%) | 8467/13827 (61.2%) |
| 7 to 13 drinks | 755/3900 (19.4%) | 747/3696 (20.2%) | 291/1679 (17.3%) | 787/4552 (17.3%) | 2580/13827 (18.7%) |
| 14 to 20 drinks | 480/3900 (12.3%) | 459/3696 (12.4%) | 159/1679 (9.5%) | 520/4552 (11.4%) | 1618/13827 (11.7%) |
| >20 drinks | 311/3900 (8.0%) | 304/3696 (8.2%) | 145/1679 (8.6%) | 402/4552 (8.8%) | 1162/13827 (8.4%) |
| **Pre-existing comorbidities** |  |  |  |  |  |
| Hypertension | 1544/4011 (38.5%) | 1750/3768 (46.4%) | 1476/1729 (85.4%) | 4217/4692 (89.9%) | 8987/14200 (63.3%) |
| Hyperlipidaemia | 910/4011 (22.7%) | 2445/3768 (64.9%) | 367/1729 (21.2%) | 3735/4692 (79.6%) | 7457/14200 (52.5%) |
| Type 2 Diabetes | 354/4011 (8.8%) | 596/3768 (15.8%) | 276/1729 (16.0%) | 1523/4692 (32.5%) | 2749/14200 (19.4%) |
| Chronic kidney disease | 458/4010 (11.4%) | 442/3767 (11.7%) | 363/1727 (21.0%) | 1031/4689 (22.0%) | 2294/14193 (16.2%) |
| Cancer | 1643/4011 (41.0%) | 1474/3768 (39.1%) | 805/1729 (46.6%) | 2130/4692 (45.4%) | 6052/14200 (42.6%) |
| Depression | 541/4011 (13.5%) | 462/3768 (12.3%) | 198/1729 (11.5%) | 575/4692 (12.3%) | 1776/14200 (12.5%) |
| Stroke | 145/4011 (3.6%) | 154/3768 (4.1%) | 92/1729 (5.3%) | 381/4692 (8.1%) | 772/14200 (5.4%) |
| **Characteristics of AMI** |  |  |  |  |  |
| Mean age at AMI (SD) | 67.5 (11.73) | 67.1 (9.94) | 73.6 (9.49) | 72.8 (8.71) | 69.9 (10.45) |
| Median length of stay (Q1; Q3) | 2.0 (1.0; 6.0) | 1.0 (1.0; 5.0) | 3.0 (1.0; 8.0) | 2.0 (1.0; 7.0) | 2.0 (1.0; 6.0) |
| **STEMI/Non-STEMI** |  |  |  |  |  |
| STEMI | 380/4011 (9.5%) | 236/3768 (6.3%) | 144/1729 (8.3%) | 315/4692 (6.7%) | 1075/14200 (7.6%) |
| Non-STEMI | 844/4011 (21.0%) | 576/3768 (15.3%) | 409/1729 (23.7%) | 945/4692 (20.1%) | 2721/14200 (19.5%) |
| Unspecified | 2799/4011 (69.8%) | 2965/3768 (78.7%) | 1181/1729 (68.3%) | 3459/4692 (73.7%) | 10404/14200 (73.3%) |
| **Complications** |  |  |  |  |  |
| Cardiac Arrest | 26/4011 (0.6%) | 19/3768 (0.5%) | 12/1729 (0.7%) | 29/4692 (0.6%) | 86/14200 (0.6%) |
| Cardiogenic Shock | 11/4011 (0.3%) | 6/3768 (0.2%) | 8/1729 (0.5%) | 18/4692 (0.4%) | 43/14200 (0.3%) |
| **Interventional management strategy** |  |  |  |  |  |
| Coronary angiogram only | 469/4011 (11.7%) | 453/3768 (12.0%) | 189/1729 (10.9%) | 605/4692 (12.9%) | 1716/14200 (12.1%) |
| Percutaneous coronary intervention | 2930/4011 (73.0%) | 3004/3768 (79.7%) | 1205/1729 (69.7%) | 3445/4692 (73.4%) | 10584/14200 (74.5%) |
| Coronary artery bypass grafting | 135/4011 (3.4%) | 271/3768 (7.2%) | 74/1729 (4.3%) | 388/4692 (8.3%) | 868/14200 (6.1%) |
| **Primary Care Engagement** |  |  |  |  |  |
| **Prior to AMI** |  |  |  |  |  |
| Primary care visits within 1 month^c^ (mean (SD)) | 0.9 (1.22) | 1.0 (1.18) | 1.2 (1.35) | 1.2 (1.36) | 1.1 (1.28) |
| Primary care visits between 2 and 6 months^d^(mean (SD)) | 4.1 (4.00) | 4.9 (3.97) | 6.5 (5.15) | 6.4 (4.56) | 5.4 (4.46) |
| **Post-AMI** |  |  |  |  |  |
| Primary care visits within 1 month^c^ (mean (SD)) | 1.4 (1.58) | 1.5 (1.37) | 1.9 (1.64) | 1.8 (1.59) | 1.6 (1.55) |
| Primary care visits between 2 and 6 months^d^ (mean (SD)) | 5.5 (5.32) | 5.9 (4.77) | 8.0 (5.89) | 7.8 (5.58) | 6.7 (5.46) |
| a. *Universal health care alone* identified by responding “No health insurance”, “Department of Veterans’ affairs white or gold care” or “Health care concession card”, *Universal health care plus additional hospital cover* indicates private health insurance without extras, *Universal health care, additional hospital and out of hospital cover* indicates Private health insurance without extras.  b. *Not working (including fully retired)* identified by responding with 0 paid work hours, selecting “Not working” or indicating a retirement age, *Part time* identified by responding between 1 and 35 hours of paid work per week, or selecting “Part time”, *Full time* identified by responding with over 35 hours for paid work per week, or selecting “Full Time”.  c. GP visits within 28 days of AMI  d. GP visits between 29 and 180 days of AMI Figure S4: Adherence at 12 months post AMI by prior treatment exposure adjusted for age, sex, income, education level, STEMI/Non-STEMI and Chronic illness 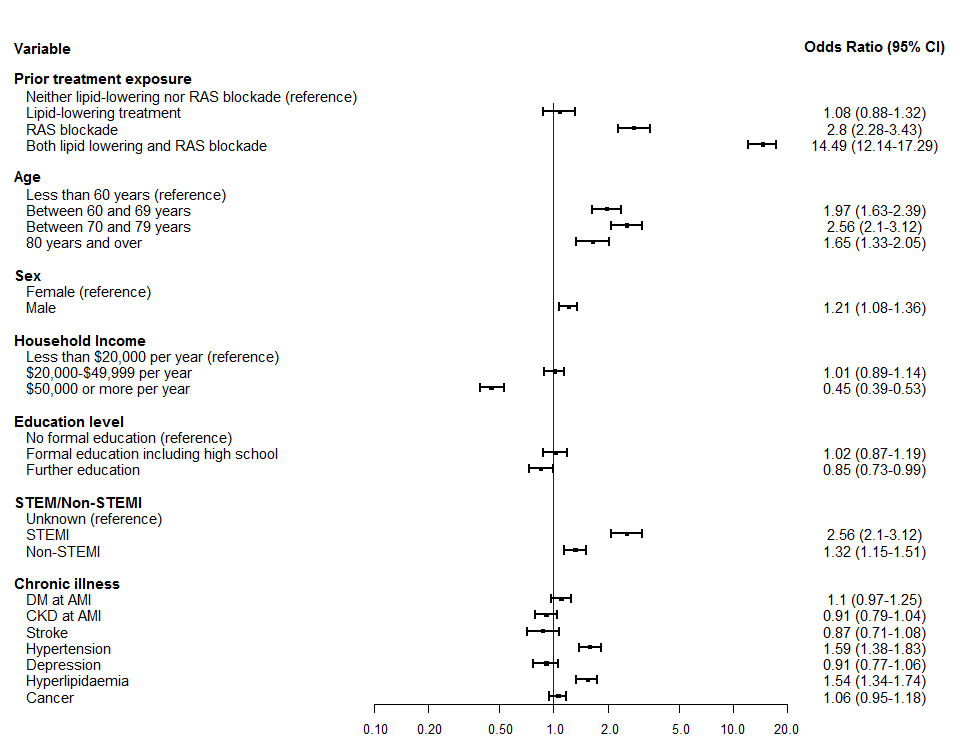 | | | | |  |

# References

1. Up Study C, Banks E, Redman S, Jorm L, Armstrong B, Bauman A, et al. Cohort profile: the 45 and up study. Int J Epidemiol. 2008;37(5):941-7.

2. Mealing NM, Banks E, Jorm LR, Steel DG, Clements MS, Rogers KD. Investigation of relative risk estimates from studies of the same population with contrasting response rates and designs. BMC Med Res Methodol. 2010;10:26.

3. McCormick N, Lacaille D, Bhole V, Avina-Zubieta JA. Validity of myocardial infarction diagnoses in administrative databases: a systematic review. PLoS One. 2014;9(3):e92286.

4. Statistics ABo. Remoteness Structure [Available from: <https://www.abs.gov.au/websitedbs/d3310114.nsf/home/remoteness+structure>.

5. WHO Collaboating Centre for Drug Statistics Methodology. Structure and principles [cited 2019 November]. Available from: <https://www.whocc.no/atc/structure_and_principles/>.

6. Elith J, Leathwick JR, Hastie T. A working guide to boosted regression trees. Journal of Animal Ecology. 2008;77(4):802-13.

7. Elith* J, H. Graham* C, P. Anderson R, Dudík M, Ferrier S, Guisan A, et al. Novel methods improve prediction of species’ distributions from occurrence data. Ecography. 2006;29(2):129-51.

8. Derksen S, Keselman HJ. Backward, forward and stepwise automated subset selection algorithms: Frequency of obtaining authentic and noise variables. British Journal of Mathematical and Statistical Psychology. 1992;45(2):265-82.

9. Smith G. Step away from stepwise. Journal of Big Data. 2018;5(1):32.

10. Thompson B. Stepwise Regression and Stepwise Discriminant Analysis Need Not Apply here: A Guidelines Editorial. Educational and Psychological Measurement. 1995;55(4):525-34.

11. Wasserstein RL, Lazar NA. The ASA Statement on p-Values: Context, Process, and Purpose. The American Statistician. 2016;70(2):129-33.

12. Friedman J, Hastie T, Tibshirani R. Additive Logistic Regression: A Statistical View of Boosting. The Annals of Statistics. 2000;28:337-407.
